# A comparative view of early development in the corals *Favia lizardensis*, *Ctenactis echinata*, and *Acropora millepora* - morphology, transcriptome, and developmental gene expression

**DOI:** 10.1186/s12862-016-0615-2

**Published:** 2016-02-29

**Authors:** Nami Okubo, David C. Hayward, Sylvain Forêt, Eldon E. Ball

**Affiliations:** Evolution, Ecology and Genetics, Bldg 46, Research School of Biology, Australian National University, Canberra, ACT 0200 Australia; ARC Centre of Excellence for Coral Reef Studies, James Cook University, Townsville, QLD 4811 Australia; Current Address: Department of Economics, Tokyo Keizai University, 1-7-34 Minamimachi, Kokubunji, Tokyo Japan

**Keywords:** Coral development, *Favia*, *Ctenactis*, *Acropora*, Transcriptome, In situ hybridization, *brachyury*, *chordin*, *forkhead*

## Abstract

**Background:**

Research into various aspects of coral biology has greatly increased in recent years due to anthropogenic threats to coral health including pollution, ocean warming and acidification. However, knowledge of coral early development has lagged. The present paper describes the embryonic development of two previously uncharacterized robust corals, *Favia lizardensis* (a massive brain coral) and *Ctenactis echinata* (a solitary coral) and compares it to that of the previously characterized complex coral, *Acropora millepora*, both morphologically and in terms of the expression of a set of key developmental genes.

**Results:**

Illumina sequencing of mixed age embryos was carried out, resulting in embryonic transcriptomes consisting of 40605 contigs for *C.echinata* (N50 = 1080 bp) and 48536 contigs for *F.lizardensis* (N50 = 1496 bp). The transcriptomes have been annotated against Swiss-Prot and were sufficiently complete to enable the identification of orthologs of many key genes controlling development in bilaterians. Developmental series of images of whole mounts and sections reveal that the early stages of both species contain a blastocoel, consistent with their membership of the robust clade. In situ hybridization was used to examine the expression of the developmentally important genes *brachyury*, *chordin* and *forkhead*. The expression of *brachyury* and *forkhead* was consistent with that previously reported for *Acropora* and allowed us to confirm that the pseudo-blastopore sometimes seen in robust corals such as *Favia* spp. is not directly associated with gastrulation. *C.echinata chordin* expression, however, differed from that seen in the other two corals.

**Conclusions:**

Embryonic transcriptomes were assembled for the brain coral *Favia lizardensis* and the solitary coral *Ctenactis echinata*. Both species have a blastocoel in their early developmental stages, consistent with their phylogenetic position as members of the robust clade. Expression of the key developmental genes *brachyury, chordin* and *forkhead* was investigated, allowing comparison to that of their orthologs in *Acropora*, *Nematostella* and bilaterians and demonstrating that even within the Anthozoa there are significant differences in expression patterns.

**Electronic supplementary material:**

The online version of this article (doi:10.1186/s12862-016-0615-2) contains supplementary material, which is available to authorized users.

## Background

Molecular phylogenetic studies show that the stony corals (Scleractinia) include two large clades, the “complex” and “robust” [1–5]. Although molecular analyses consistently support this dichotomy, morphological support based on adult anatomy is lacking. However, each clade has a characteristic pattern of embryonic development [6, 7]. In most of the complex corals which have been studied there is either no, or only a minimal, blastocoel prior to gastrulation, giving rise to the term “prawn chip” for the flattened cellular bilayer that is present in the genus *Acropora* [8–10]. In contrast to the complex corals, robust corals form an obvious blastocoel before gastrulation (reviewed in [7]). Commonly, these blastocoel stage embryos develop a depression in their surface and resemble in shape gastrulating embryos of complex corals. However, at this stage they are composed of a single cell layer surrounding the blastocoel; they subsequently resume a spheroidal shape before forming a blastopore and undergoing gastrulation.

We recently used in situ hybridization to characterize the expression of *Acropora millepora* orthologs of several genes that play key roles in bilaterian gastrulation and axis formation including *brachyury, chordin and forkhead* [11]. Brachyury is a member of the T-box transcription factor family which in vertebrates is expressed around the blastopore at gastrulation, then in involuting mesoderm and finally in the notochord (reviewed in [12]). Studies in diverse organisms (e.g. mouse [13, 14] and ctenophore [15, 16]) suggest that its universal role is in the regulation of genes involved in cell adhesion and the control of morphogenetic movements.

Chordin is the product of the vertebrate ortholog of the *Drosophila* short gastrulation gene. It is an antagonist of BMP2/4; *chordin* and *bmp2/4* genes are involved in determining the dorsal/ventral axis in all bilaterians, but with an axis inversion unique to chordates [17, 18].

Fox (or Forkhead) genes encode a large and ancient family of transcription factors which is united by the presence of the winged helix domain. Members of the FoxA subfamily have diverse functions including acting as “pioneer” transcription factors which facilitate the remodelling of chromatin and the actions of nuclear receptors, with their many roles. In mammals FoxA genes are important in the development of several endodermally derived organs including lung and liver (reviewed in [19]).

**Fig. 1:**
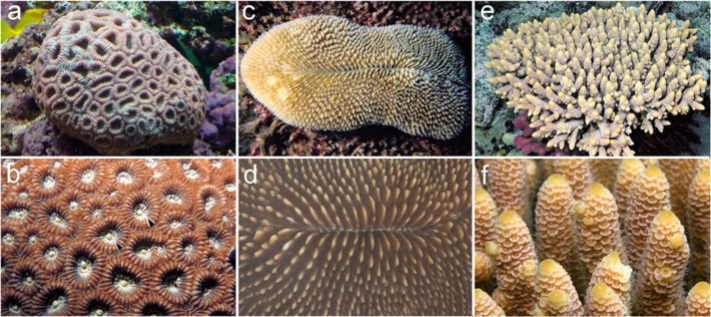
Comparative adult morphology of the species studied. (**a**) Small colony of *F.lizardensis*. (**b**) A closeup view shows that each of the circular or oblong structures surrounds a single polyp (arrowheads). (**c**) *C.echinata* is a solitary coral, consisting of a single large polyp. (**d**) Closeup of the central portion showing the slit from which the polyp emerges to feed. (**e**) The *A.millepora* colony is composed of many branches emerging from a single base. (**f**) Closeup of a few branches, each covered in many polyps. Each scale-like structure on the branch marks the location of a single polyp. Photo credits with thanks to: (**a**) Lawrence Cope, (**b**, **c**, **e**) Australian Institute of Marine Science, (2015). AIMS Coral Fact Sheets: (**b**) [47], (**c**) [48], (**e**) [49], (**d**) Nami Okubo, (**f**) Zoe Richards

In the present study we first describe the early embryonic development of two robust coral species with very distinctive adult morphologies and previously uninvestigated developmental morphologies, *Favia lizardensis* and *Ctenactis echinata. F.lizardensis* is a massive brain coral (Fig. 1a, b), *C.echinata* is a solitary coral (Fig. 1c, d) and the previously characterised *A.millepora* is a staghorn coral (Fig. 1e, f). We then used RNASeq and de novo assembly to generate transcriptome assemblies from *C.echinata* and *F.lizardensis* embryos and identified orthologs of all three of the genes previously characterised in *A. millepora*. This information allowed us to carry out in situ hybridizations revealing both conserved and novel patterns of gene expression.

## Methods

### Embryo collection

Several specimens of *F.lizardensis* and *C.echinata* were collected near the Sesoko Marine Biological Laboratory in Okinawa, Japan (26°38′ N, 127°52′ E) in July, 2009. They were brought to the laboratory and placed in tubs before the predicted time of spawning. *C.echinata* spawned from 23:00–24:00 on July 14, and different pairs of *F.lizardensis* colonies spawned on July 20, 21 and 22, 2009. Gametes of each species were gently stirred to mix the bundles and ensure fertilization. Fertilized eggs were placed into 2.5 L containers in filtered sea water and development allowed to proceed. For observation, approximately 50 eggs or embryos were placed in a 75-mm Petri dish under a light microscope. The water temperature was maintained at 26.0 to 26.5 °C throughout the period of observation and culture. Staged embryos were harvested and either snap frozen in liquid nitrogen, and stored at −80 deg C until used for nucleic acid extraction, or fixed for 20–40 min in 3.7 % formaldehyde in filtered HEPES buffered seawater (pH 8.0), and stored in 100 % methanol at −20 deg C until further treatment.

Adult coral colonies were collected with permission of the Agriculture, Forestry and Fisheries Department of the Okinawa Prefectural Government. No permit is required for collecting coral embryos in Japan. Permission numbers for *F.lizardensis* and *C.echinata* are “21–22”. Embryos were imported into Australia under CITES permit T-AG-09-500096 where the studies were carried out.

### Morphology

Further observations were made and photos taken of embryos fixed with paraformaldehyde and stored in methanol at −20 deg C. Embryos of both species are sufficiently large to allow freehand cutting with a microknife; such sections are often more informative than embedded, sectioned material, as the plane of section is more easily determined.

### RNA isolation

Total RNA was prepared using Tri Reagent (Ambion) based on the method of Chomczynski and Sacchi [20]. Total RNA was prepared from *F.lizardensis* gastrula stage embryos. For *C.echinata*, total RNA was prepared separately from fertilized eggs, morula, and gastrula stage embryos. Equal amounts of RNA from each of these stages were pooled. Poly (A) + RNA was prepared using the Dynabeads mRNA purification kit (Life Technologies).

### High-throughput sequencing

Libraries were prepared for 76 bp paired end sequencing with the mRNA-Seq Sample Prep Kit (Illumina) using 100 ng poly(A) + RNA. The *F.lizardensis* library was sequenced by Macrogen Inc, Seoul, South Korea. The *C.echinata* library was sequenced at the Biomolecular Resource Facility, JCSMR, ANU. Sequences were trimmed with libngs [21] using a minimum length of 40 bp and minimum quality score of 15. The assemblies were generated with the Trinity assembler (r2013-12-16) [22], with default parameters, discarding contigs shorter than 200 bp. After assembly, redundant sequences were removed using cdhit-est (4.6.1) [23] with a minimum similarity of 90 % and a word size of 8 bp.

To assess the quality of the transcriptome assemblies, reciprocal blast searches were carried out with proteome databases from the sea anemone *Nematostella vectensis* (downloaded from UniProt [24]) and the coral *A.millepora* [25] using an evalue cutoff of 10^−6^. The transcriptomes were also searched with the CEGMA 248 eukayotic core genes dataset [26] using tblastn with an evalue cutoff of 10^−10^. The coverage of these genes in the transcriptomes was measured by dividing the length of the alignment of the top blast hit by the query length. The transcriptomes were annotated using the top hits of blastx searches (evalue cutoff 10^−6^) against the UniProtKB/Swiss-Prot database [27].

### cDNA and PCR

First strand cDNA was made from total RNA using PrimeScript reverse transcriptase (Takara Bio) and an oligo d(T) primer (oligo d(T)20VN) according to the manufacturer’s protocol. Candidate target transcripts were identified in the transcriptome assemblies with local blast (NCBI) and PCR primers were designed manually or using MacVector (MacVector, Inc). PCR was carried out using Platinum Taq DNA Polymerase High Fidelity (Life Technologies). PCR products were purified using the QIAquick PCR Purification Kit (Qiagen) and ligated into pGEM-T Easy (Promega). Plasmids were sequenced with vector and internal primers using Big Dye Terminator v3.1 (Applied Biosystems) and reactions were run on an ABI 3730 at the Biomolecular Resource Facility, JCSMR, ANU. Sequence analyses and alignments were carried out using DNASTAR (Lasergene) and MacVector (MacVector Inc).

### In situ hybridization

Digoxygenin (DIG) labelled RNA probes were made from linearized plasmid templates. Hybridization conditions were as described in [28]. DIG labelling was detected with an alkaline phosphatase conjugated anti-DIG antibody (Roche) using BCIP/NBT substrate mix (Vector SK-5400). Embryos were dehydrated and cleared through a glycerol dehydration series, mounted on microscope slides in 90 % glycerol and digital images were captured with a Spot camera mounted on a Wild Photomakroskop. Digitized images were processed using Adobe Photoshop.

## Results

### Morphological comparisons

**Fig. 2:**
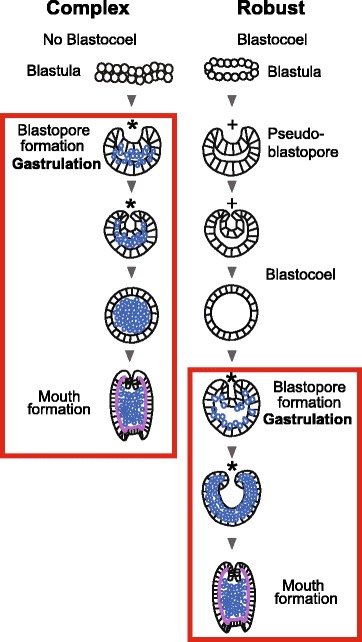
Schematic diagram showing the two major types of scleractinian development leading up to gastrulation. The complex corals typically have a minimal blastocoel before the start of gastrulation and only assume a spherical shape once gastrulation is complete. The robust corals, in contrast, develop a pseudo-blastopore (+) and pass through a hollow spheroidal stage before gastrulation begins. Endoderm = blue circles, mesoglea = purple lines, asterisk = blastopore. Modified from [6, 7]

**Fig. 3:**
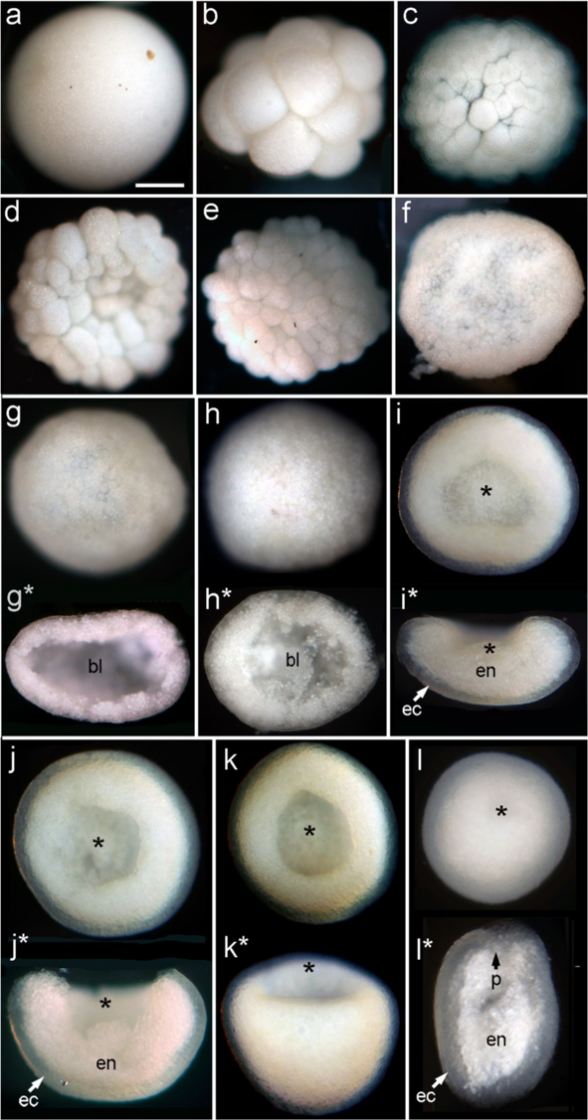
*F.lizardensis* embryonic development. (**a-f**) illustrate successive stages of development with micrographs of wholemounts. (**g**, g*)-(**l**, l*) are paired images of single embryos, with the oral wholemount view above and the vertical section or side view (k*) below. (**a**) Egg. (**b-e**) The developing embryo undergoes successive stages of cell division while maintaining a basically spherical structure. (**f**) Cell division has continued as the embryo begins to flatten. (**g**, g*) At this stage the embryo consists of a spheroidal blastula containing a large blastocoel (bl). (**h**, h*) Gastrulation has begun, with presumptive endoderm moving into the blastocoel (bl). (**i**, i*) A wide blastopore (asterisk in this and succeeding panels) is now apparent in the wholemount and yolk-laden endoderm (en, in this and succeeding panels) fills the shallow bowl formed by the ectoderm (ec, in this and succeeding panels). (**j**, j*) The embryo begins to assume a more rounded shape. (**k**, k*) Wholemount blastoporal and side views. (**l**, l*) In this planula the blastopore (now the oral pore) is much less apparent, and the section reveals that the planula has elongated and the oral ectoderm is bending axially to form the pharynx (p). Scale = 100uM

**Fig. 4:**
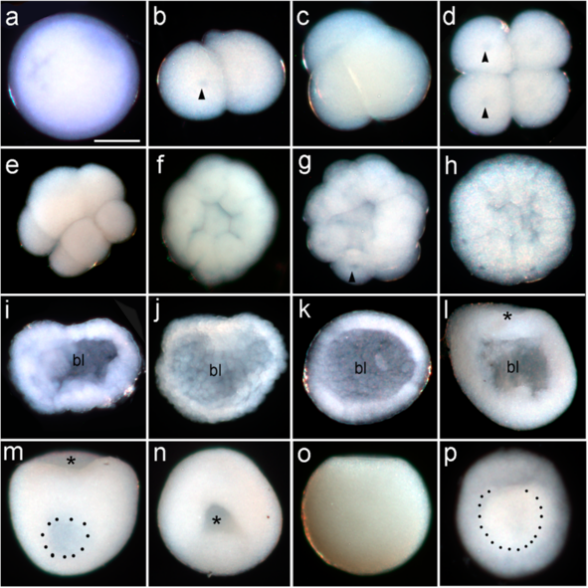
*C.echinata* embryonic development. (**a**) Egg. (**b-e**) Successive cell divisions result in an eight-celled embryo. Nuclei are apparent as darker areas within the cells (arrowheads in **b**,**d**,** g**). (**f-h**) Further cell divisions lead to production of a spheroidal embryo. (**i**) Section of an embryo of similar age to that in (**h**) showing an obvious blastocoel (bl, in this and succeeding panels). (**j**-**k**) Sections showing the embryo forming a hollow sphere. (**l**) The sphere then elongates in the oral-aboral axis, as gastrulation begins with cells moving inward into the blastocoel (asterisk marks the blastopore in this and succeeding panels). (**m-n**) Lateral and oral views of a single whole mount embryo. The remaining blastocoel is apparent as a darker area in (**m**), while the blastopore (asterisk) is shown in (**n**) . (**o**) The late gastrula stage, which has now become ciliated and capable of swimming. (**p**) Early planula stage-the whiter central area (outlined with black dots) marks the extent of the yolk-filled endoderm. Scale bar = 100um

The basic features of gastrulation in robust and complex corals are compared in Fig. 2. The major difference between the two groups is that the robust corals pass through a greater number of distinctly recognizable morphological stages, several of which have an obvious blastocoel, before gastrulation occurs. In some species this includes formation of a temporary depression, which we have termed a pseudo-blastopore [6], before a hollow spheroidal shape is resumed leading into true gastrulation. The morphological development of *F.lizardensis* is shown in Fig. 3, and that of *C.echinata* in Fig. 4. Detailed descriptions are given in the figure captions. Note that there is a considerable size difference between comparable stages of the three species. For example, diameters of fixed eggs are: *A.millepora* (470 um), *F.lizardensis* (390 um), and *C.echinata* (260 um); other stages maintain approximately the same size ratios.

### Transcriptomes

RNA prepared from embryonic stages of *C.echinata* and *F.lizardensis* was processed for Illumina sequencing. After removal of low quality data (length <40 bp or quality score <15), 18 million paired end reads (2.8 Gbp), and 23 million paired end reads (3.5 Gbp) were obtained for *C.echinata* and *F.lizardensis,* respectively. After assembly, this resulted in 40605 contigs (28 Mbp) for *C.echinata*, and 48536 contigs (40 Mbp) for *F.lizardensis* (Table 1).

**Table 1:** Transcriptome assembly details

	Reads	Contigs	Mean length (nt)	N50 (nt)	Longest transcript (nt)
*Ctenactis echinata*	18506158	40605	708	1080	11258
*Favia lizardensis*	22361373	48536	834	1496	16006

The contig size distribution is shown in Additional file 1. While the average (mean) contig lengths are 708 bp and 834 bp for *C.echinata* and *F.lizardensis* respectively, indicating that the size distribution is weighted towards smaller sized contigs, the N50 value is 1080 bp for *C.echinata* and 1496 bp for *F.lizardensis*. These values are similar to, or larger than, those for several other short read coral transcriptome assemblies [29–32].

The quality of the assemblies was assessed in a number of ways. Blast searches were carried out using proteomes from two cnidarian species, *N. vectensis* (24435 sequences) [33] and *A.millepora* (26622 sequences) [25] (Additional file 2). Approximately 50 % of the *F.lizardensis* contigs and 56 % of the *C.echinata* contigs had hits (evalue <10^−6^) in the *A.millepora* proteome. The values for searches against the *N.vectensis* proteome were 46 and 40 % for *C.echinata and F.lizardensis,* respectively. In reciprocal searches, approximately 79 % of sequences in the *A.millepora* proteome had hits in the *C.echinata* transcriptome and 84 % had hits in the *F.lizardensis* transcriptome. For *N.vectensis*, the values were 76 and 77 % for searches against *C.echinata and F.lizardensis,* respectively. The higher values achieved by searching the transcriptome sequences with the proteome sequences is not unexpected since many of the assembled transcriptome sequences are short, and are likely to represent non-coding regions or incomplete open reading frames. The transcriptomes were also searched with a highly conserved set of 248 core eukaryotic proteins [34, 35]. Over 98 % of these were identified in both transcriptomes. The length of the best match was compared to the query length to assess the coverage of the core proteins in the transcriptomes (Additional file 3). The majority of the core proteins were covered by more than 80 % in both transcriptomes. Finally, to annotate the transcriptomes, the assemblies were searched against the UniProtKB/Swiss-Prot database [27] using blastx (evalue < 10^−6^). 39.4 % (16007 contigs) of the *C.echinata* contigs and 32.5 % (15716 contigs) of the *F.lizardensis* contigs could be annotated (Additional files 4 and 5).

### Developmental gene sequences

Blast searches were carried out for developmentally important sequences associated with gastrulation and axis determination and orthologous to ones previously characterized in *Acropora*. Orthologs of *brachyury*, *forkhead* and *chordin* were identified in both the *C.echinata* and *F.lizardensis* transcriptomes. Alignments of the predicted proteins with those from *A.millepora* and *N.vectesis* are shown in Additional files 6, 7 and 8. The *C.echinata* and *F.lizardensis* sequences share a higher level of amino acid identity with each other than they do with the *A.millepora* sequences, consistent with their phylogenetic relationships. To isolate template sequences for RNA probe production, PCR primers were used to amplify products from embryonic cDNA (Additional files 9 and 10).

### Gene expression patterns

**Fig. 5:**
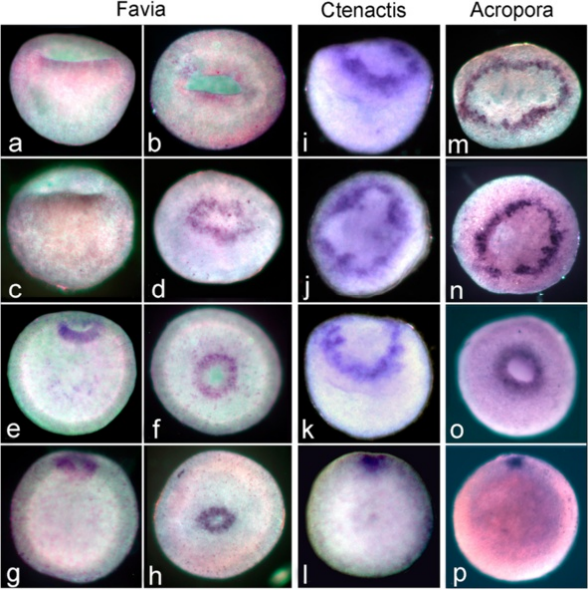
*Brachyury* expression compared in the 3 species. The youngest embryos are at the top of the figure and the oldest at the bottom. In *F.lizardensis*, age matched embryos are shown in lateral view on the left and oral view on the right. The earliest expression seen is on the rim of the bowl-shaped embryo surrounding the blastopore (**a**-**b**). Expression continues around the blastopore/oral pore, as it closes (**c**-**h**). *C.echinata* expression (**i**-**l**) develops in a similar way, first appearing on the rim of the bowl-shaped embryo, with expression continuing as the blastopore gradually closes. In *A.millepora* (**m**-**p**)*,* the general pattern is similar, with *Ambra* expression appearing around the blastopore as it forms at the end of the prawn chip stage (**m**) and persisting as the blastopore gives rise to the oral pore (**p**)

**Fig. 6:**
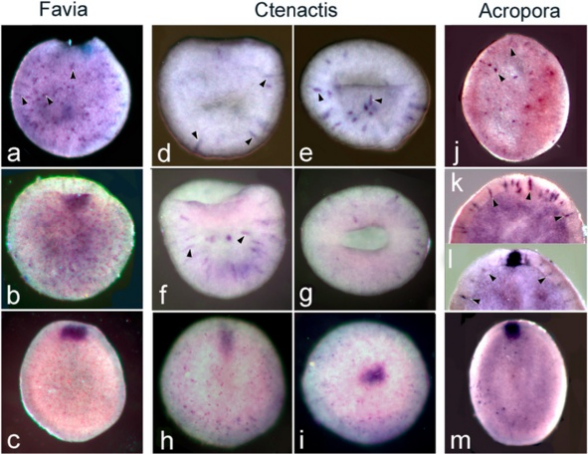
*Forkhead* expression compared in the three species, each of which is arranged in order of age. (**a**) In *F.lizardensis* the earliest expression is in scattered ectodermal cells (arrowheads) as the blastopore is closing. (**b**) A little later in development an area of contiguous expression appears in cells on the rim of the blastopore. (**c**) Expression continues in the oral pore as the sphere elongates to become an early planula. In *C.echinata* expression appears at a comparable or slightly earlier stage, once again in isolated cells in the ectoderm (arrowheads). The left column (**d**, **f**, **h**) shows embryos in side view, while the right (**e**, **g**, **i**) shows the same embryos in blastoporal view (i.e. **d** and **e**, **f** and **g**, and **h** and **i** are micrographs of the same embryo). (**j-m**) In *A.millepora* the same pattern of expression is followed. (**j**) This embryo shows expression only in isolated ectodermal cells (arrowheads). (**k**) Portion of an embryo showing magnified view of scattered ectodermal cells which are expressing. (**l**) Oral end of another embryo showing expression in the blastopore/oral pore and in scattered ectodermal cells. (**m**) Early planula larva showing expression in the oral pore, only a few isolated ectodermal cells continue to express

**Fig. 7:**
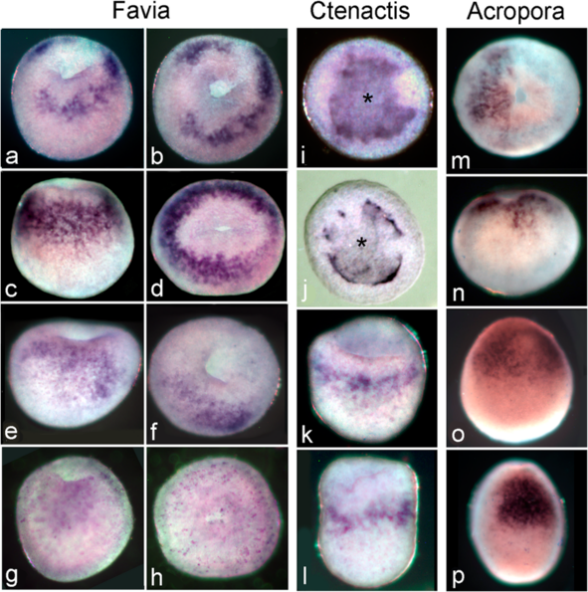
*Chordin* expression in the three species. (**a-h**) Expression of *Flchd* in three embryos of similar age, and one that is slightly older, in side (**a**,**c**,**e**,**g**) and oral views (**b**, **d**, **f**, **h**). Expression becomes more localized and then fades away as the embryo ages. *C.echinata* *chordin* shows a different early expression pattern from that seen in the other species, being expressed in the presumptive endoderm at the blastula stage, as shown in (**i**), which is looking down onto the developing blastopore. (**j**) As the presumptive endoderm sinks aborally to form the blastopore expression becomes localized to its margins, as shown in this blastoporal view. In (**i, j**) the center of the developing blastopore is marked with an asterisk. (**k**) Lateral view of a slightly older embryo showing expression in the ectodermal wall of the bowl-shaped gastrula. (**l**) Expression in the ectoderm of the wall continues as the embryo continues to elongate. In *A.millepora*, expression begins in isolated cells at the oral end surrounding the blastopore [11] before becoming localized to one side of the blastopore (**m**) where expression gradually intensifies. In the early planula expression is localized to a patch of ectoderm on one side toward the oral end (**o**). This patch of expression then becomes stronger and more restricted (**p**), before disappearing well before settlement

Figures 5, 6 and 7 compare patterns of embryonic gene expression in the three species.

#### *Brachyury* expression

In both *F.lizardensis* (Fig. 5a-h) and *C.echinata* (Fig. 5i-l) the earliest *brachyury* expression occurs on the rim of the bowl-shaped gastrula as it is elongating in the future oral-aboral axis and the central depression is deepening. This circular expression pattern is similar to the expression of * A.millepora brachyury* (*Ambra*), which appears as the embryo is rounding up at the end of the prawn chip stage (Fig. 5m, see also [11]). In all species and stages expression is limited to a circle around the blastopore, which steadily decreases in circumference as the blastopore closes.

#### *Forkhead* expression

The earliest *forkhead* expression in all three species occurs in isolated ectodermal cells as the blastopore is closing (arrowheads in Fig. 6a, d, e, f, j, k, l) with these cells apparent slightly earlier in *C.echinata* relative to blastopore closure (Fig. 6d, e). Expression then appears in the ectoderm surrounding the closing blastopore/opening oral pore (Fig. 6c, h, i, m). 

#### *Chordin* expression

Expression of *chordin* in *F.lizardensis* is variable in extent. The embryos shown in Fig. 7a-f are all similar in age, yet the oral views (Fig. 7b, d, f) reveal considerable differences in *chd* expression; a half circle plus a separate patch of expression (Fig. 7b), a complete circle around the closing blastopore with one area of weaker expression (Fig. 7d), and asymmetric expression on one side of the blastopore (Fig. 7f, g), which becomes weaker as the blastopore closes (Fig. 7g, h).

*Chordin* expression in *C.echinata* first appears over a broad area of tissue on one side of the flattened blastula, corresponding to the area which will invaginate to form the endoderm (Fig. 7i). Slightly later, as the presumptive endoderm moves aborally relative to the surrounding ectoderm, the general expression fades and becomes limited to the inner margins of the rim of the bowl-shaped structure that is being created (Fig. 7j). As the embryo continues to elongate in the oral/aboral axis the ring of ectodermal expression is shifted aborally, away from the rim of the bowl (Fig. 7k, l). Expression continues as a ring in the ectodermal walls of a vase-shaped structure, even as the embryo elongates to form a planula (Fig. 7l).

In *A.millepora*, *chordin* expression begins in scattered cells around the blastopore [11] before becoming localized to one side (Fig. 7m). Slightly later expression becomes more widespread, both in terms of the number of cells and their distribution (Fig. 7n, o). As the spherical embryo elongates in the oral/aboral axis to form a planula larva, expression is limited to a patch of oral ectoderm covering approximately one-third of the circumference of the embryo (Fig. 7p). Expression disappears entirely by the time of settlement (not shown).

## Discussion

The only genomic/transcriptomic resource available for corals closely related to the two studied here is a transcriptome constructed from two adult *Favia* of undetermined species from the Red Sea [31]. Here we report embryonic transcriptome assemblies for *C.echinata* and *F.lizardensis*. A comparison with other cnidarian datasets and with the CEGMA database indicates that these assemblies present a comprehensive picture of early developmental transcriptomes in these species.

In all recent coral phylogenies, the genera *Favia* and *Ctenactis* are grouped among the robust corals, well separated from the complex corals such as *Acropora*. Unsurprisingly, this is reflected in the genes studied here, which are usually regarded as being highly conserved. This is especially true of *brachyury* and *forkhead*, which have long stretches of identical sequence for the three corals. Not only this, but orthologous genes in the three corals are more similar to each other than they are to their *N.vectensis* orthologs, again consistent with phylogeny. It is clear that *chordin* is the least conserved of the genes considered here, in both sequence and expression.

In addition to being the best non-scleractinian sequence comparator for corals, *N.vectensis* is also the most relevant comparator for expression patterns and for function, so those two topics will be discussed together below.

*NvBra* expression surrounds the blastopore in *N.vectensis* [36, 37] as does its ortholog in *A.millepora,* where it demarcates the ectoderm from the presumptive endoderm; the latter defined by the expression of *Amsnail* [11, 38]*.* This expression is consistent with the role of *brachyury* in the ctenophore *Mnemiopsis,* where functional tests have demonstrated that it is involved in the regulation of the morphogenetic movements involved in gastrulation [16] and in the mouse, where mutant studies have demonstrated that the primitive streak is condensed and thickened [14, 39]. In both cases Brachyury is believed to affect cell movements by regulating the expression of cell adhesion genes [13, 16]. In the robust corals also, *brachyury* is associated with the blastopore during gastrulation. The association of early *brachyury* expression with the start of gastrulation is confirmed by comparison to the wholemounts and sections shown in Figs. 3 and 4. Figure 4k in particular shows that blastula stage *C.echinata* form a hollow sphere before the beginning of gastrulation, in contrast to *A.millepora*, which only assumes a spherical shape near the completion of gastrulation (Fig. 5o, p)

In *N.vectensis, forkhead* is expressed around the blastopore in a pattern very similar to that of *brachyury*. Indeed, Fritzenwanker et al. [40] described the two genes as “an evolutionarily ancient synexpression group in Eumetazoa”, while Magie et al. [41] hypothesized that *N.vectensis* FoxA defined the ectoderm/endoderm boundary on the basis of double in situs. However, in a more recent study Röttinger et al. [37] demonstrated that *brachyury* expression starts earlier than that of *FoxA* in *N.vectensis*, as is the case in *A.millepora*, where the expression of *forkhead* (*Amfkh*) in the blastopore/oral pore occurs later than that of *Ambra*, toward the end of gastrulation [11]. Expression in *A.millepora* is also seen in scattered ectodermal cells either before or in association with the start of expression around the blastopore [11] (Fig. 6). The expression of *forkhead* in the robust corals is similar to that in *A.millepora,* in both scattered ectodermal cells, and around the blastopore. Examination of the sequences shown in Additional file 7 reveals that the coral sequences are very highly conserved, while that of *N.vectensis* shows some significant divergence. These observations raise the possibility that we are dealing with a paralogous sequence, rather than a true ortholog, in *N.vectensis*. However, no other *N.vectensis* sequence gives as good a match as the one shown. Another possibility for explaining the differing expression in the two species is the co-option of the coral *forkhead* gene to a second function in single cells.

The important patterning role of chordin as an antagonist of BMP2/4 has been well characterized in several bilaterians (reviewed in [17, 18]) as well as in *N.vectensis*. The expression and role of *chordin* in *N.vectensis* have been reported [37, 42–45]. The most surprising finding of these studies is that *Nvbmp2/4* and *Nvchd* are expressed at the same end of the directive axis [42, 44], which is orthogonal to the oral-aboral axis, in contrast to their deployment in bilaterians. Genikhovich et al. [46] have recently built on previous work to argue that the interaction between chordin and BMP2/4 is critical in determining the zones of expression of other genes involved in patterning the directive axis of *N.vectensis*. They conclude that it is the positioning of chordin that is critical and that the maximum of BMP2/4 signalling will always be opposite the chordin expression domain regardless of the spatial expression pattern of BMP2/4. The earliest *chordin* expression pattern reported for *N.vectensis* is a ring surrounding the area where the blastopore will form [37], while the earliest reported by Rentzsch et al. in *N.vectensis* [42] and Hayward et al. in *A.millepora* [11] is in the form of scattered cells centered on the blastopore prior to its closure. Slightly later, but still before blastopore closure, this expression becomes localized to one side of the blastopore. Then, as the planula forms, this expression becomes localized to an ectodermal patch toward the oral end in both organisms. There is some variability in the expression of *F.lizardensis chordin* (*Flchd*). Some specimens show a complete or partial ring around the blastopore, while in others there is asymmetric expression in a localized patch on one side of the embryo. It is unclear whether this variability represents different stages of a rapidly changing expression pattern or is due to a lesser staining sensitivity in some individuals. In *C.echinata*, *chordin* expression appears earlier, at the blastula stage in a zone corresponding to the region which will invaginate. As gastrulation proceeds this generalized expression fades, remaining intense only at the border of the invaginating region (Fig. 7j). While there may be some asymmetry in the expression pattern at this stage, as gastrulation continues and the central depression deepens, expression takes the form of a complete ring around the blastopore with no sign of asymmetry. Since restricted localization of chordin expression is critical to axial patterning in embryonic *Nematostella,* the apparent lack of restricted localization in *C.echinata* is puzzling. It is possible that the *C.echinata* embryos available to us may all be younger than a presumed critical period or that such a critical period occurred between the ages of embryos available to us. A more comprehensive in situ hybridization series combined with functional studies will be required to resolve this question.

## Conclusions

The solitary coral *Ctenactis echinata* and the brain coral *Favia lizardensis*, like other robust corals, have a well developed blastocoel before gastrulation. Assembly of embryonic transcriptomes for both species facilitated the isolation of sequences corresponding to key developmental genes. Expression of *brachyury, chordin* and *forkhead* was investigated, allowing comparison to that of their orthologs in *Acropora* and *Nematostella*. All three corals exhibit early *forkhead* expression in scattered ectodermal cells, a pattern that has not been reported in *N. vectensis.* In the developmental stages of *C.echinata* and *F.lizardensis* that we were able to examine the expression of *chordin* does not show the overt asymmetry seen in *Acropora* and *Nematostella*. The conserved association of *brachyury* expression with the blastopore, in addition to the morphological studies, confirms that the early concavity in the blastula stage of robust corals (the pseudo-blastopore) does not indicate the onset of gastrulation. The expression of forkhead (FoxA) in isolated ectodermal cells, as well as around the blastopore, in all corals so far examined and in contrast to *Nematostella,* as well as the very different expression of *Ctenactis chordin,* accentuate the need for further comparative studies of cnidarian development if we are to achieve a comprehensive understanding of the evolution of this process.

### Availability of supporting data

The data are available from the NCBI TSA database; Project ID PRJNA297488.

## Additional files

Additional file 1:
**Distribution of assembled contig lengths.** (PDF 328 kb) Additional file 2:
**Reciprocal blast searches with** ***Acropora*** **and** ***Nematostella*** **proteomes.** (PDF 393 kb) Additional file 3:
**Coverage of 248 core eukaryotic proteins in the** ***C.echinata*** **and** ***F.lizardensis*** **transcriptomes.** The human set of the core protein dataset was used for the analysis. (PDF 395 kb) Additional file 4:
**C.echinata transcriptome annotation.** Results of blastx search (e < 10^−6^) against the UniProtKB/Swiss-Prot database. (CSV 2894 kb) Additional file 5:
***F.lizardensis*** **transcriptome annotation.** Results of blastx search (e < 10^−6^) against the UniProtKB/Swiss-Prot database. (CSV 2857 kb) Additional file 6:
**Brachyury alignment.** A. Alignment of brachyury sequences from: *AmBra*, *Acropora millepora*, KJ914894; *Flbra*, *Favia lizardensis*, this study; *Cebra*, *Ctenactis echinata*, this study; *Nvbra*, *Nematostella vectensis*, AAO27886. The Tbox domain region is outlined in red. B. Percentage amino acid identities between the sequences. Percentage amino acid similarities are shown in brackets. (PDF 1935 kb) Additional file 7:
**Forkhead alignment.** A. Alignment of forkhead sequences from: *Amfkh*, *Acropora millepora*, JT020561; *Flfkh*, *Favia lizardensis*, this study; *Cefkh*, *Ctenactis echinata*, this study; *Nvfkh*, *Nematostella vectensis*, XP_001634555. The forkhead superfamily domain is outlined in red. B. Percentage amino acid identities between the sequences. Percentage amino acid similarities are shown in brackets. (PDF 1505 kb) Additional file 8:
**Chordin alignment.** A. Alignment of chordin sequences from: *Amchd*, *Acropora millepora*, JR985644; *Flchd*, *Favia lizardensis*, this study; *Cechd*, *Ctenactis echinata*, this study; *Nvchd*, *Nematostella vectensis*, XP_001633548. The two chordin domains are outlined in red. B. Percentage amino acid identities between the sequences. Percentage amino acid similarities are shown in brackets. (PDF 2911 kb) Additional file 9:
**PCR primers.** The sequences of PCR primers used to amplify products from first strand cDNA. (DOCX 47 kb) Additional file 10:
**Fasta file of sequences of PCR products.** (TXT 7 kb)
