# Supplementary figures and images for: A comparative view of early development in the corals Favia lizardensis, Ctenactis echinata, and Acropora millepora - morphology, transcriptome, and developmental gene expression
Source: BMC Evol Biol. 2016 Feb 29;16:48. doi: 10.1186/s12862-016-0615-2 (PMC4770532; doi:10.1186/s12862-016-0615-2)

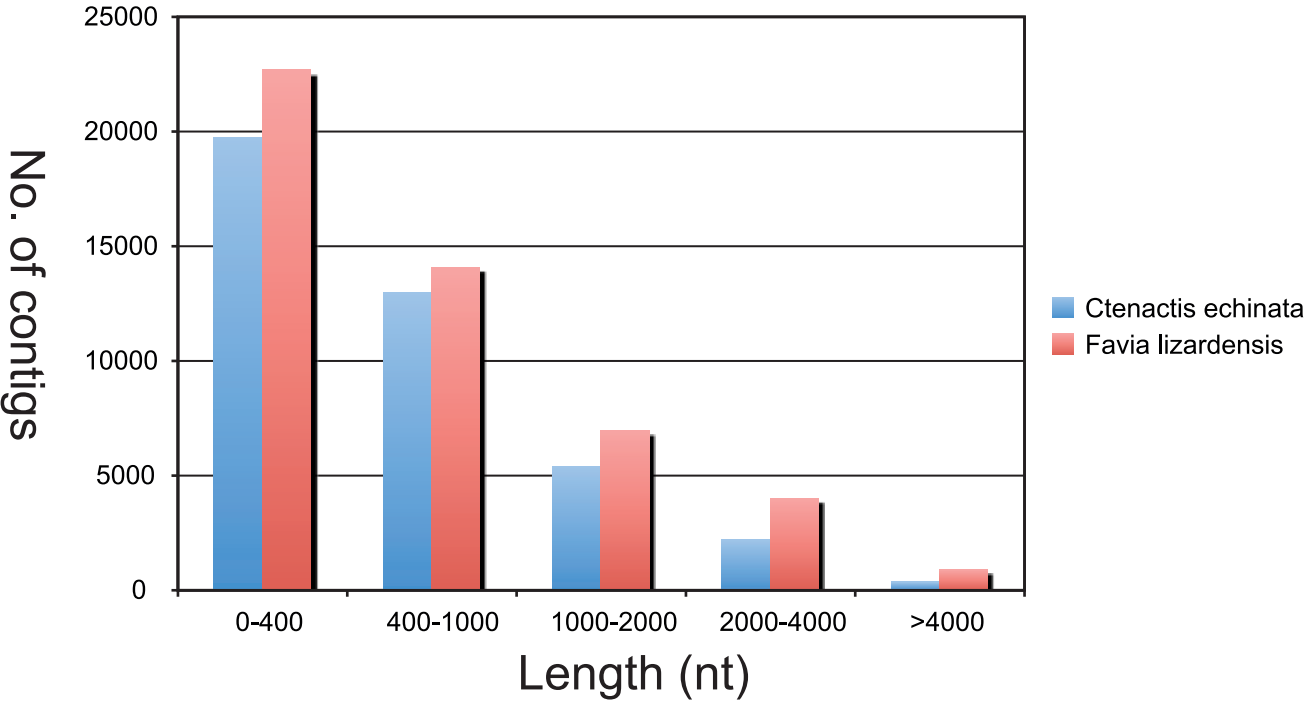

Distribution of contig lengths

Supplement: Additional file 1: — Distribution of assembled contig lengths. (PDF 328 kb) [file 12862_2016_615_MOESM1_ESM.pdf]
