# Supplementary material for: A comparative view of early development in the corals Favia lizardensis, Ctenactis echinata, and Acropora millepora - morphology, transcriptome, and developmental gene expression
Source: BMC Evol Biol. 2016 Feb 29;16:48. doi: 10.1186/s12862-016-0615-2 (PMC4770532; doi:10.1186/s12862-016-0615-2)
Supplement: Additional file 2: — Reciprocal blast searches with Acropora and Nematostella proteomes. (PDF 393 kb) [file 12862_2016_615_MOESM2_ESM.pdf]

Additional File 2

A

	A.millepora	*N.vectensis*
C.echinata	56.2	46.1
F.lizardensis	49.6	39.4

Percentage of contigs with hits ($e<10^{-6}$) in *A.millepora* and *N.vectensis* proteomes

B

	C.echinata	*F.lizardensis*
A.millepora	78.7	84.1
N.vectensis	75.6	76.6

Percentage of sequences in *A.millepora* and *N.vectensis* proteomes with hits ($e<10^{-6}$) in *C.echinata* and *F.lizardensis* transcriptomes
