# Supplementary material for: A comparative view of early development in the corals Favia lizardensis, Ctenactis echinata, and Acropora millepora - morphology, transcriptome, and developmental gene expression
Source: BMC Evol Biol. 2016 Feb 29;16:48. doi: 10.1186/s12862-016-0615-2 (PMC4770532; doi:10.1186/s12862-016-0615-2)
Supplement: Additional file 3: — Coverage of 248 core eukaryotic proteins in the C.echinata and F.lizardensis transcriptomes. The human set of the core protein dataset was used for the analysis. (PDF 395 kb) [file 12862_2016_615_MOESM3_ESM.pdf]

Additional File 3

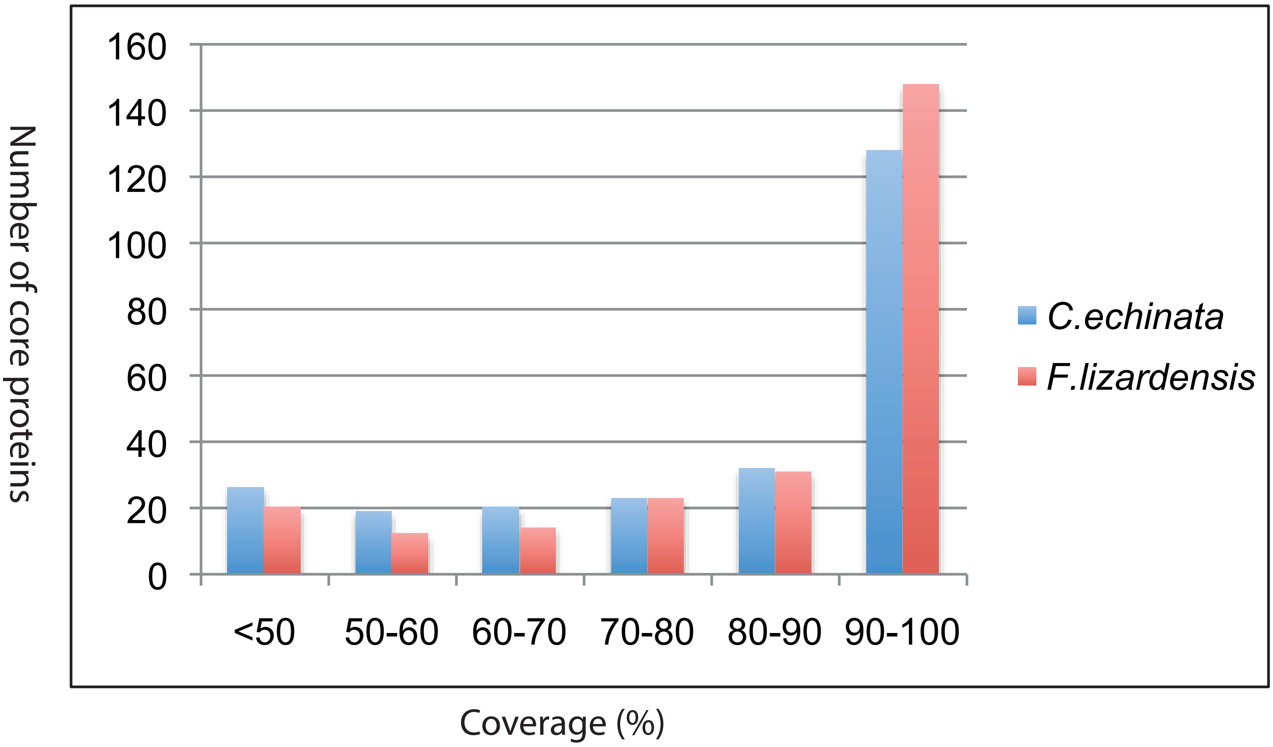

Coverage of 248 core eukaryotic proteins in the *C.echinata* and *F.lizardensis* transcriptomes. The human set of the core protein datatset was used for the analysis.
