# Supplementary material for: A comparative view of early development in the corals Favia lizardensis, Ctenactis echinata, and Acropora millepora - morphology, transcriptome, and developmental gene expression
Source: BMC Evol Biol. 2016 Feb 29;16:48. doi: 10.1186/s12862-016-0615-2 (PMC4770532; doi:10.1186/s12862-016-0615-2)
Supplement: Additional file 6: — Brachyury alignment. A. Alignment of brachyury sequences from: AmBra, Acropora millepora, KJ914894; Flbra, Favia lizardensis, this study; Cebra, Ctenactis echinata, this study; Nvbra, Nematostella vectensis, AAO27886. The Tbox domain region is outlined in red. B. Percentage amino acid identities between the sequences. Percentage amino acid similarities are shown in brackets. (PDF 1935 kb) [file 12862_2016_615_MOESM6_ESM.pdf]

Additional File 6 Brachyury alignment

A

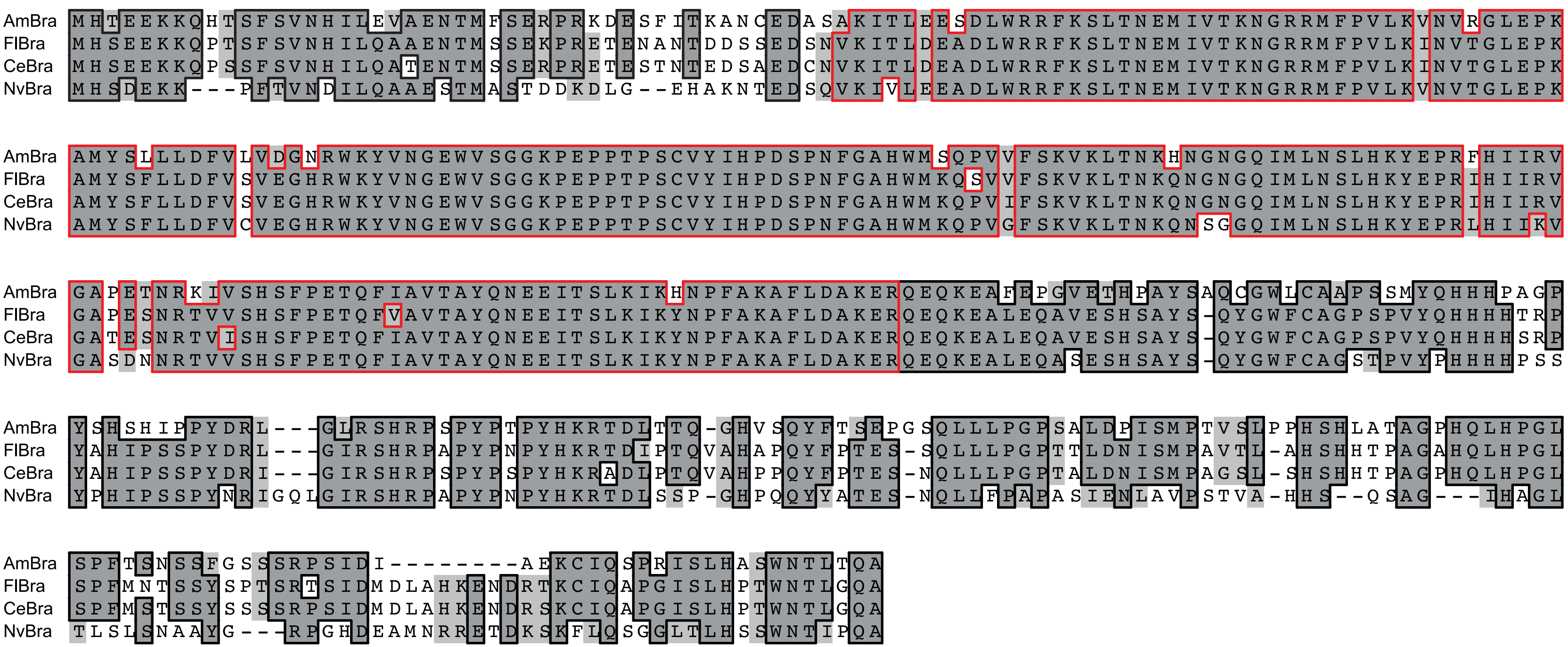

B

	Acropora	*Ctenactis*	*Favia*	*Nematostella*
Acropora		76% (8%)	75% (8%)	65% (12%)
Ctenactis			92% (4%)	71% (12%)
Favia				70% (12%)
Nematostella				

Alignment length: 403-406 amino acids
