# Supplementary material for: A comparative view of early development in the corals Favia lizardensis, Ctenactis echinata, and Acropora millepora - morphology, transcriptome, and developmental gene expression
Source: BMC Evol Biol. 2016 Feb 29;16:48. doi: 10.1186/s12862-016-0615-2 (PMC4770532; doi:10.1186/s12862-016-0615-2)
Supplement: Additional file 7: — Forkhead alignment. A. Alignment of forkhead sequences from: Amfkh, Acropora millepora, JT020561; Flfkh, Favia lizardensis, this study; Cefkh, Ctenactis echinata, this study; Nvfkh, Nematostella vectensis, XP_001634555. The forkhead superfamily domain is outlined in red. B. Percentage amino acid identities between the sequences. Percentage amino acid similarities are shown in brackets. (PDF 1505 kb) [file 12862_2016_615_MOESM7_ESM.pdf]

Additional File 7 forkhead alignment

A

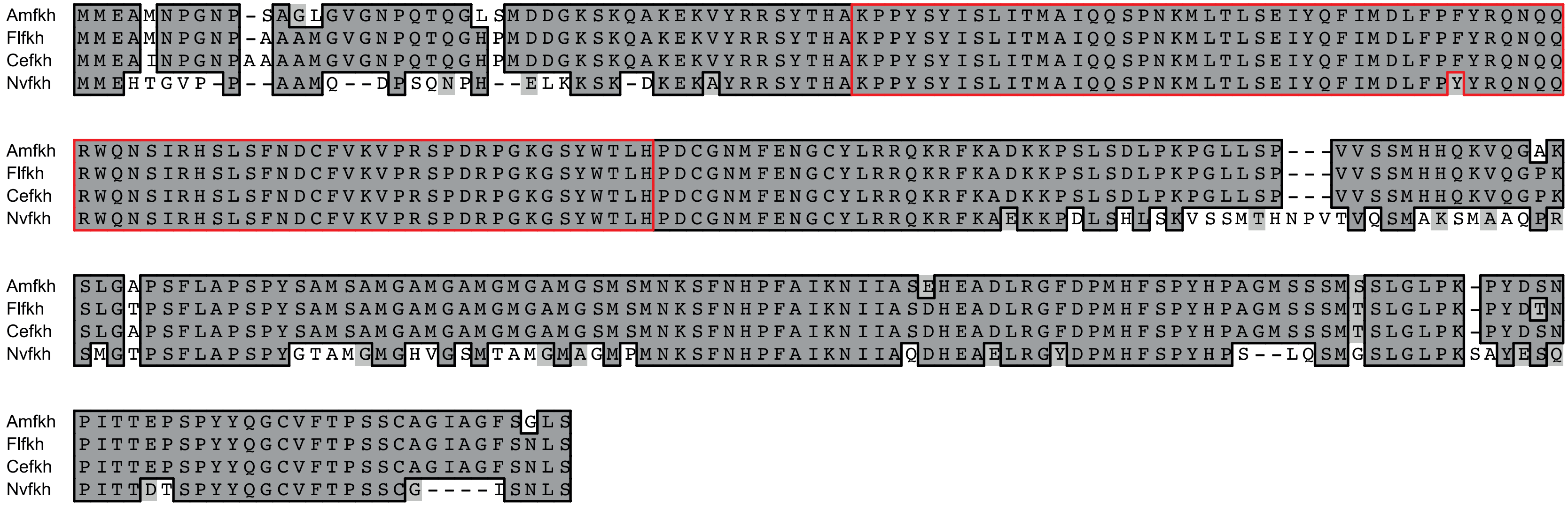

B

	Acropora	*Ctenactis*	*Favia*	*Nematostella*
Acropora		96% (2%)	96% (2%)	70% (7%)
Ctenactis			98% (1%)	71% (6%)
Favia				71% (6%)
Nematostella				

Alignment length: 295-300 amino acids
