# Supplementary material for: A comparative view of early development in the corals Favia lizardensis, Ctenactis echinata, and Acropora millepora - morphology, transcriptome, and developmental gene expression
Source: BMC Evol Biol. 2016 Feb 29;16:48. doi: 10.1186/s12862-016-0615-2 (PMC4770532; doi:10.1186/s12862-016-0615-2)
Supplement: Additional file 8: — Chordin alignment. A. Alignment of chordin sequences from: Amchd, Acropora millepora, JR985644; Flchd, Favia lizardensis, this study; Cechd, Ctenactis echinata, this study; Nvchd, Nematostella vectensis, XP_001633548. The two chordin domains are outlined in red. B. Percentage amino acid identities between the sequences. Percentage amino acid similarities are shown in brackets. (PDF 2911 kb) [file 12862_2016_615_MOESM8_ESM.pdf]

A

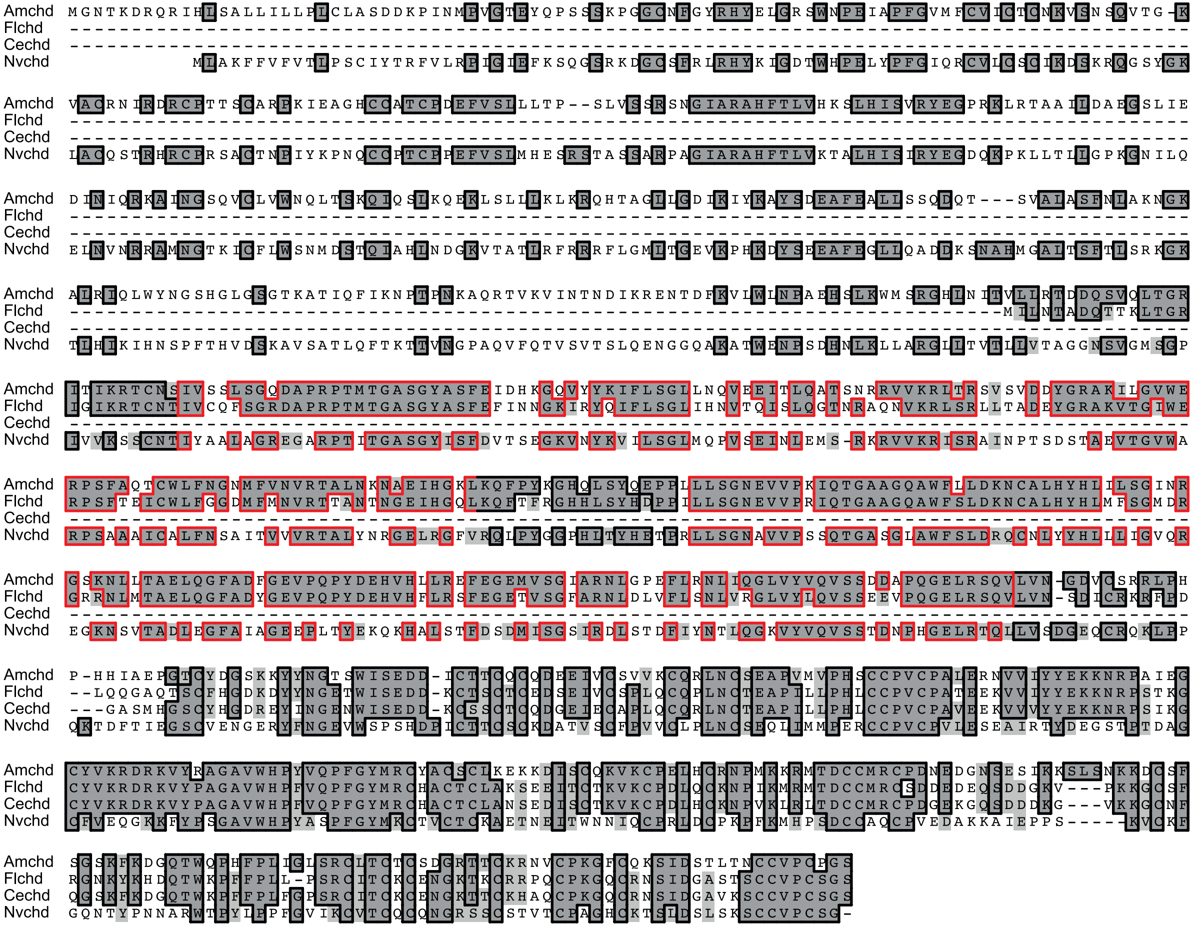

B

	Acropora	Ctenactis	Favia	Nematostella
Acropora		65% (12%)	62% (12%)	39% (15%)
Ctenactis			79% (8%)	40% (21%)
Favia				41% (18%)
Nematostella				

Alignment length: 236-240amino acids

Additional File 8

chordin alignment
