# Supplementary material for: A comparative view of early development in the corals Favia lizardensis, Ctenactis echinata, and Acropora millepora - morphology, transcriptome, and developmental gene expression
Source: BMC Evol Biol. 2016 Feb 29;16:48. doi: 10.1186/s12862-016-0615-2 (PMC4770532; doi:10.1186/s12862-016-0615-2)
Supplement: Additional file 9: — PCR primers. The sequences of PCR primers used to amplify products from first strand cDNA. (DOCX 47 kb) [file 12862_2016_615_MOESM9_ESM.docx]

Additional File 9

| Primer | Sequence |
| --- | --- |
| Flfkh_F1 | CGCTGTTGAGGACGAAAGAGTAATC |
| Flfkh_R2 | CTCAATGTTATGGTTCTCTTAAG |
| FlBra_F1 | CAAGTCGCTGACCAACGAGATG |
| FlBra_R1 | GGCTGGAGTGTGATGAGAATGC |
| Flchd_F1 | GATGATACTGAACACCGCTGACC |
| Flchd_R2 | TTCTCCCCCTTGCTTCTTTACC |
| Cefkh_F1 | GAAAGAGAAAGTTTACCGCCGC |
| Cefkh_R1 | TGAATCGTAAGGTTTAGGCAGTCC |
| CeBra_F1 | AAAGCAACCCTCATCGTTTTCC |
| CeBra_R1 | AGCCAGGGTGAAGTTGATGAGG |
| Cechd_F1 | GGTTTCCTCATTTACAGCAGGGAG |
| Cechd_R1 | AGCAATCTATGGAGGGGCTACG |

PCR primer sequences
